# Dynamic diagnostic relationism: a new diagnostic paradigm for complex rapidly changing clinical conditions

**DOI:** 10.1186/1754-9493-8-21

**Published:** 2014-05-07

**Authors:** Lawrence A Lynn

**Affiliations:** 1 The Sleep and Breathing Research Institute, 1275 Olentangy RR, Columbus, Ohio 43212, USA

**Keywords:** Threshold, Diagnostics, Sepsis, Biomarker, Critical, Complex, Science, Relationism, Reductionist

## Abstract

Decades of large, apparently well-designed clinical trials have failed to generate reproducible results in the investigation of many complex rapidly evolving and changing conditions such as sepsis. One possibility for the failure is that 20th century threshold science may be too simplistic to apply to complex rapidly changing conditions, especially those with unknown times of onset. There is an acute need to reconsider the fundamental validity of the application of simple threshold science in the study of complex rapidly evolving and changing conditions. In this letter, four potential axioms are presented which define a new science which assesses the probability of disease as a function of motion images of all the available clinical data.

## Letter to the editor

*“Everything should be made as simple as possible, but not simpler”. *Albert Einstein

Threshold science using receiver operating characteristics (ROC) was developed by the British to detect German planes flying over the English Channel during the London blitz of 1940–41. Since the late 1970s, threshold science and ROC have been increasing employed in the field of medical diagnostics [1,2]. However, unlike German planes which maintained the same morphology as they crossed the channel, many clinical conditions such as sepsis rapidly change in relational morphology over time as they progress toward a fatal conclusion. While the ROC may perform well for simple infection related decisions, [3] it may not be sufficient with more complex clinical data sets such as those generated during sepsis. This may explain why, over the past 20 years, threshold science [1,2] has failed to generate reproducible results in the study of rapidly changing and cascading conditions such as sepsis [4]. Considerable speculation has arisen to explain the lack of reproducibility [5,6]. One possibility for the failure is that threshold science and the ROC, despite an illustrious pedigree dating back to the battle of Britain, are too simplistic to apply to complex, rapidly evolving biologic conditions, especially those with unknown times of onset [7]. Four axioms of medical diagnostics are presented to encourage reconsideration of the fundamental reliance on threshold science in the investigation of rapidly evolving and changing clinical conditions such as sepsis.

Axiom 1

The probability of a complex, rapidly evolving condition existing in relation to a measured biomarker value for this condition is a function of the time of onset of the rapidly progressive condition in relation to the time of biomarker sampling.

This axiom is easily understood by considering the complex, rapidly evolving condition of sepsis. As noted above the receiver operating characteristics curve (ROC) [1], which is widely used in threshold science lacks the dimension of time and is therefore poorly applicable to biomarkers which vary over time since the time of onset of sepsis is generally unknown. To solve the problem of the static nature of the ROC, researchers commonly use a “fixed” time for all cases, such as “time of arrival to ER”, “time of occurrence of a rapid response team call”, or the “time when sepsis is first suspected”. While these times may appear consistent, they have no consistent relationship to the onset or sepsis or the infection itself.

Axiom 2

The probability of a complex, rapidly evolving condition existing in relation to a measured biomarker value for this condition is a function the values of the relational data and cannot be determined by using only the measured biomarker value and the pretest probability.

It is common to see ROC analysis of biomarkers applied in isolation. This is inaccurate since the clinical evaluation of complex, rapidly evolving condition such as sepsis never includes only one variable. An analogy would be to assess the probability of a good hand when one is dealt an ace in five card draw poker. This might be of some interest to a mathematician, but it would always be wrong to take action based on this probability because the player always receives five cards which must be considered in combination to assess the probability (strength) of the hand. For example a WBC of 15 would have a completely different sensitivity and specificity for sepsis in isolation than when combined with a platelet count of 104, an immature neutrophil percentage of 9, and venous bicarbonate of 18. Therefore in rapidly progressing cascading conditions it is the peri-test probability and not simply the pretest probability which must be included in the analysis. The peri-test probability is affected by all the relevant preceding and present data available at the time the test becomes available.

Axiom 3

The probability of a complex, rapidly evolving condition existing in relation to a measured biomarker value for this condition is a function of the probability of the trajectory (the time series pattern) of the biomarker.

A third problem with conventional method of ROC analysis of a biomarker value is the lack of consideration for the trajectory of the probability of disease. Axiom 3 may, in some respects, appear similar to axiom 1, but has a different basis and implications. For example, an absolute neutrophil count which is rapidly rising generates a different probability for sepsis than the single value of its last measurement even if additional absolute neutrophil values are unknown. Except, perhaps, in quantum theory, probabilities are not affected by failing to “look”. The fact that the player has not looked at the cards which he or she has been dealt after the first card (or considered the results of sequential or additional tests), does not affect the probability of the dynamically dealt hand. Rather each new card generates a new aggregate probability and the dealing process generates a time-series of probabilities with a trajectory. This trajectory of probability might be called the “biomarker trend probability”. The probability derived from the absolute neutrophil count is therefore a pattern over time or a “time-series of probabilities” regardless of whether serial absolute neutrophil count values are determined or considered.

Of course, when axiom 3 is combined with axiom 2, it is clear that that any given biomarker trend (like any given biomarker value) can never be considered in isolation. Rather all the biomarker trends must be considered together relationally to fulfill both axiom 2 and 3. Finally, combining axioms 1–3 renders a 4th axiom of medical diagnostics for rapidly evolving clinical conditions.

Axiom 4

The available probability of a condition at any given time is a function of the probability of the motion image of all the relevant data available at that time.

## Conclusion

It is time to reconsider the fundamentals of the science of rapidly evolving and changing critical illness such as sepsis. Static threshold based definitions and even definitions which include trends over time may not be sufficient. These four axioms demonstrate the weaknesses of simple threshold science of the 1980s in the setting of complex rapidly evolving clinical conditions. These weaknesses may explain the lack of reproducibility of large clinical trials of conditions, such as sepsis. By examining the fundamentals of threshold science a new science of relational dynamic diagnostics may emerge.

## Competing interest

Dr. Lynn has issued and pending patents which relate to the detection of sepsis and other clinical conditions by imaging time-matrix distortions. He is the owner of Lyntek Medical Technologies which develops sepsis detection software. He is the co-inventor of PateintStormTracker, visualization software for imaging and presentation of time-matrix distortions. He has licensed patents for monitor based pattern recognition to Covidien.

## Author information

LAL is a pulmonary and critical care physician and sepsis investigator. He serves as executive director of the Sleep and Breathing Research Institute in Columbus Ohio. He also has served on the FDA standards committee for pulse oximetry monitoring.

## References

[B1] Metz CE Basic principles of ROC analysis Semin Nucl Med 1978 4 283 298 112681 10.1016/s0001-2998(78)80014-2

[B2] Pauker SG Kassirer JP The threshold approach to clinical decision making N Engl J Med 1980 302 20 1109 1117 10.1056/NEJM198005153022003 7366635

[B3] Wigton RS Connor JL Centor RM Transportability of a decision rule for the diagnosis of streptococcal pharyngitis Arch Intern Med 1986 146 1 81 83 10.1001/archinte.1986.00360130103014 3510600

[B4] Opal SM Dellinger RP Vincent JL Masur H Angus DC The next generation of sepsis clinical trial designs: what is next after the demise of recombinant human activated protein C? Crit Care Med 2014 Apr 8. [Epub ahead of print] 10.1097/CCM.0000000000000325 PMC4135726 24717456

[B5] Lynn LA The diagnosis of sepsis revisited - a challenge for young medical scientists in the 21st century Patient Saf Surg 2014 8 1 10.1186/1754-9493-8-1 24383420 PMC3944929

[B6] Vincent JL Opal SM Marshall JC Tracey KJ Sepsis definitions: time for change Lancet 2013 381 9868 774 775 10.1016/S0140-6736(12)61815-7 23472921 PMC4535310

[B7] Lynn LA Curry JP Patterns of unexpected in-hospital deaths: a root causes analysis Patient Saf Surg 2011 5 1 3 10.1186/1754-9493-5-3 21314935 PMC3045877

